# Genome-wide association study to identify biological and metabolic pathways associated with carcass portion weights in turkeys^☆^^☆☆^: GWAS OF TURKEY CARCASS PORTION WEIGHTS

**DOI:** 10.1016/j.psj.2025.105194

**Published:** 2025-04-18

**Authors:** Emily M. Leishman, Ryley J. Vanderhout, Benjamin J. Wood, Christine F. Baes, Shai Barbut

**Affiliations:** a Department of Animal Biosciences, University of Guelph, Guelph, Ontario N1G 2W1, Canada; b Department of Animal and Veterinary Sciences, Aarhus University, Tjele 8830, Denmark; c Hybrid Turkeys, Suite C, 650 Riverbend Drive, Kitchener, Ontario N2K 3S2, Canada; d School of Veterinary Science, University of Queensland, Gatton, Queensland 4343, Australia; e Institute of Genetics, Vetsuisse Faculty, University of Bern, Bern 3001, Switzerland; f Department of Food Science, University of Guelph, Guelph, Ontario N1G 2W1, Canada; g Adaptation Physiology, Wageningen University, Wageningen 6700, the Netherlands

**Keywords:** Carcass, Genetics, Meat, Meleagris gallopavo, Poultry, Breeding

## Abstract

The application of genetic and genomic improvement strategies in the poultry industry has been widely successful at improving meat yield and efficiency, however some challenges persist. As demand for larger and leaner birds increases, we have not fully assessed how selection for growth affects various carcass portions. The objective of this study was to conduct a genome wide association study (**GWAS**) and functional analysis on turkey carcass portion weights. Phenotypic data consisted of carcass portion weights (fillets, tenders, drums, thighs) obtained at processing (*N* = 646 – 1,478). Genotypic records were available from a proprietary 65 K single nucleotide polymorphism (**SNP**) chip. A linear mixed model was used to estimate SNP effects and a 30-SNP sliding window approach was used. Across all traits, 14 functional candidate genes (**FCGs**) were identified, and these were predominately associated with protein metabolism and immune function. Interestingly, carcass portions did not share FCGs, except for the thighs and drums, which shared one functional candidate gene (PDGFB). These results add to the understanding of the genetic architecture of carcass portion weights, and this could be applied in a turkey breeding program.

## Introduction

The global demand for poultry products, particularly turkey, has demonstrated consistent growth, primarily due to the lean composition and high protein content of turkey meat (Barbut and Leishman, 2022). This demand can also be attributed to the characteristics of the product such as affordability, acceptability (i.e., no religious restrictions), and nutritional content. To meet demand, advancements have been made in turkey growth, efficiency, and meat yield, mostly stemming from improvements in genetic selection, management practices, and nutrition (Kálmán and Szőllősi, 2023). These improvements have led to turkeys that reach market weight faster and yield more meat on processing (Vanderhout et al., 2024). Turkey meat can be marketed for sale to consumers as whole carcasses, or be broken down and sold as uncooked portions or further processed (i.e., deli meat, turkey bacon). Breast fillet meat is the most valuable carcass portion; however, breast tenders, thighs, drums, and wings also have significant value. Purchasing meat in portions, further processed or as ready-to-eat products, has become increasingly popular as consumers seek convenient nutritious products. Expanding research efforts to include these less-studied portions could provide opportunities to enhance their quality and market value, thereby maximizing the overall economic potential of turkey production.

Genome wide association studies (**GWAS**) in quantitative trait loci (**QTL**) mapping have been applied across numerous livestock species and in poultry, for example, to improve body weight gains in chickens (Dadousis et al., 2021; Dou et al., 2022). In swine, a GWAS approach has been used to identify genes associated with different meat cut proportions (Xie et al., 2023). Xie et al. (2023) uncovered different candidate genes associated with the region of the body where the meat cut originated, enhancing the potential for targeted selection for optimal growth/meat shape in different parts of the body. Understanding the causative genes for traits of interest allows for the identification of major genomic regions (loci) and therefore has the potential to improve gene-based selection (Abdalla et al., 2022). Abdalla et al. (2022) identified 37 genes associated with growth and metabolism in a large population of purebred turkeys

Such analyses are not only interesting from an economic point of view (i.e., optimizing meat production, increasing economic value, etc.), but could also be interesting from an animal health and welfare perspective. The focus on breast meat yield can potentially have consequences on leg health and walking ability because of the negative relationship between breast muscle size and leg strength. Leg strength and walking ability are highly correlated with livability; it may, therefore, be beneficial to investigate the growth of other carcass portions to develop strategies for more structurally sound birds, ultimately enhancing livability. The opportunity for targeted selection to improve leg muscle development, as well as that of breast muscles, may help confer some advantages for leg health. However, analyses of the genetic architecture of carcass portion traits are lacking in turkeys compared to other species. Consequently, the aim of this study was to identify genes associated with carcass portion weights in turkeys using a GWAS approach. The approach can offer valuable insights to further understanding the physiological impacts on each of the carcass portions when predominately selecting for growth.

## Materials and methods

### Animals

Male purebred turkeys (20-24 weeks old) from three genetic lines (A, B, and C) were processed over a 44-week period. The three genetic lines consisted of two dam lines and one sire line. Of the two dam lines, Line A was selected predominately for growth and reproduction. The other dam line, Line B was selected for reproductive traits. The sire line, Line C was selected mainly for growth and meat yield. All birds were reared under identical housing conditions and management protocols (Hybrid Turkeys, 2020). Later during standard commercial processing, the birds were electrically stunned, exsanguinated, scalded, defeathered, and eviscerated before being chilled (40 min in 5°C water, 1.5–2 h in 1–2°C water, and remainder of time layered in ice). After 24 hr of chilling, carcasses were deboned and weighed. Weights were taken for four carcass portions: tenders, fillets, drums, and thighs. All protocols complied with the guidelines of the Canadian Council on Animal Care and were approved by the University of Guelph Animal Care Committee (AUP 3782).

### Phenotypic and genotype data

Summary statistics for each of the carcass portions is shown in Table 1. Genotypes were collected using a proprietary 65 K single nucleotide polymorphism (**SNP**) array (65,000 SNP; Illumina, INC.). PLINK software (Purcell et al., 2007) was used as a quality check and to remove any SNP markers that were identified in a non-autosomal region, with a minor allele frequency (<0.05), a call rate of lower than 90 %, or proportions that significantly deviated from Hardy Weinberg (*p* < 1e^-8^). Following the quality check, analysis continued with the remaining 54,407 markers.

**Table 1:** Summary statistics (mean ± standard deviation) for live weight, slaughter age, and carcass portion weights for each genetic line, as well as pedigree and genotype descriptions.

	Line A	Line B	Line C
Live weight (kg)	21.8 ± 1.666	19.2 ± 1.269	24.6 ± 1.617
Carcass portion weight (kg)			
Fillets (*N* = 659 – 1,431)^1^	4.47 ± 0.490	4.06 ± 0.420	5.13 ± 0.629
Tenders (*N* = 660 – 1,426)	0.83 ± 0.090	0.77 ± 0.073	1.01 ± 0.112
Thighs (*N* = 667 – 1,478)	3.00 ± 0.258	2.51 ± 0.208	3.44 ± 0.286
Drums (*N* = 646 – 1,460)	2.34 ± 0.199	1.94 ± 0.154	2.59 ± 0.207
Mean processing age (days)	150 ± 2.10	154 ± 3.23	144 ± 3.89
Number of animals in the pedigree	6,530	4,146	6,063
Number of genotyped birds	963	477	1,185
Number of genotyped sires	126	153	183
Number of genotyped dams	623	354	583
Number of SNP markers		54,407	

1 N = number of birds, range represents variation in sample size across the genetic lines.

### Statistical analysis

A linear mixed model was used to estimate variance components through restricted maximum likelihood carried out using the BLUPf90 family of programs (Misztal et al., 2018). The linear mixed model used is described as follows:

y=Xb+Za+e

where **y** is the portion weight, **b** is a vector of fixed effects including genetic line (3 levels: A, B, and C), hatch week-year (58 levels), age at slaughter (7 levels; 141 to 163 days); **a** is a vector of additive genetic effects distributed as **a** ∼ *N*(0, $H\sigma_{a}^{2}$), where **H** is the combined pedigree-genomic relationship matrix as described in Aguilar et al. (2010) constructed using the PREGSf90 program and $\sigma_{a}^{2}$ is the additive genetic variance; **e** is the vector of residual effects which has a distribution of **e** ∼ *N*(0, $\sigma_{e}^{2}$) where $\sigma_{e}^{2}$) is the residual variance; and **X** and **Z** are design matrices relating the observations to the fixed and random effects, respectively.

Estimates of SNP effects were derived from the estimated genomic breeding values (**gEBV**) following Wang et al. (2012), using a weighted genomic relationship matrix:

$$\hat{g}=\text{DZ}\prime {\left[\text{ZDZ}\prime \right]}^{-1}{\hat{u}}_{g}$$

where $\hat{g}$ is a vector of SNP marker effects; **D** is a diagonal matrix of weights for variances of SNPs; **Z** is a matrix relating genotype of each locus; and ${\hat{u}}_{g}$ is the vector of gEBV. A 30-SNP sliding window approach was used to identify potential genomic regions associated with carcass portion weights that may not otherwise be detected due to low variance explained by single SNPs.

### Functional analysis

Identified markers in the 99^th^ percentile of variance explained were considered significant (Vanderhout et al., 2024). Using the GALLO package in R (Fonseca et al., 2020), positional candidate genes within ±50 kb of the significant SNP from the Turkey 5.1 assembly (Dalloul et al., 2010) were retrieved using the Ensembl Genes database version 104 (https://useast.ensembl.org/Meleagris_gallopavo/Info/Index). Potential candidate genes were identified through WebGestaltR R package and *Gallus gallus* database (Wang et al., 2023). From the identified positional candidate genes, gene ontology (**GO**) enrichment analysis was performed, identifying cellular components (**CC**), molecular functions (**MF**), and metabolic pathway analyses (Kyoto Encyclopedia of Genes and Genomes (**KEGG**) database) (Kanehisa and Goto, 2000).

## Results and discussion

### Estimation of genetic parameters

Across genetic lines, heritability for portion weight was estimated as 0.47 ± 0.050 for fillets, 0.50 ± 0.048 for tenders, 0.46 ± 0.048 for thighs, and 0.64 ± 0.051 for drums. In turkeys, the heritability of breast meat yield has been reported as 0.30 (Aslam et al., 2011) and breast weight as 0.35 (Havenstein et al., 1988). Our estimates are higher than those previously reported, potentially due to the use of purebred animals and changes in genetic and environmental factors since the previous estimates. Additionally, since there were not always enough animals in each genetic line to run separate models, the lines were included in the same model with line as a fixed effect. It is possible that this may have resulted in some overestimation of genetic variance (Table 2) and the results may have been different if the lines were run separately with a larger dataset. Additionally, we assessed the two breast muscles independently (fillet vs. tender) which might make a direct comparison with past estimates challenging. However, Vanderhout et al. (2022) used the same dataset to estimate the heritability of whole breast weight (fillet + tender) and reported an estimated heritability of 0.46. Havenstein et al. (1988) also estimated heritabilities for thigh and drum weights in male and female turkeys as 0.12 – 0.17 and 0.30 – 0.44, respectively. In broiler chickens, the heritability of leg (thigh + drum) weight has been estimated as 0.33 + 0.03 (Gaya et al., 2006). The present heritability estimates are substantially higher, which could be attributed to the factors mentioned above. Overall, the estimates presented here indicate that there may be potential to select for the development of other carcass portions, like the legs. Including the weight of the thighs and/or drums in a selection program for turkeys may help balance the growth of the lower limbs against the growth of the breast muscles, considering the negative genetic correlations between breast meat yield and the weight of the leg muscles reported in Vanderhout et al. (2022). Future studies should investigate the relative benefits of larger leg size in terms of liveability indicators like leg health and walking ability.

**Table 2:** Genetic and residual variance (± standard error) for the carcass portion weights.

Trait	Genetic variance	Residual variance
Fillets	0.93962E-01 ± 0.94298E-02	0.11013 ± 0.52613E-02
Tenders	0.30103E-02 ± 0.30653E-03	0.39626E-02 ± 0.17923E-03
Thighs	0.24280E-01 ± 0.24976E-02	0.31097E-01 ± 0.14310E-02
Drums	0.18464E-01 ± 0.15636E-02	0.15208E-01 ± 0.78553E-03

### Significant SNP and positional candidate genes

**Fig. 1:**
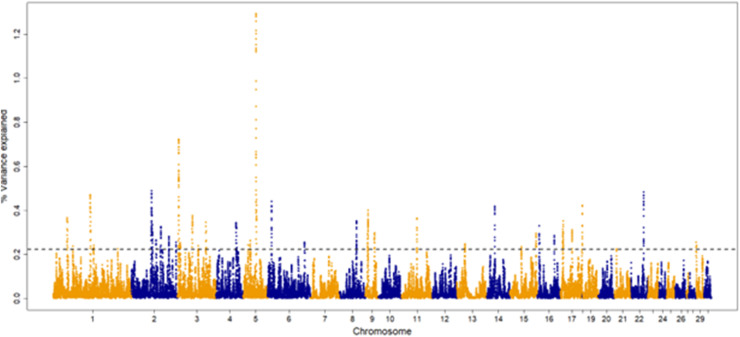
Manhattan plot for percentage of variance explained by a 30-SNP sliding window across the genome for turkey fillet weight (kg). The top 1 % of SNP windows that explain the most genetic variance are located above the horizontal dashed line.

**Fig. 2:**
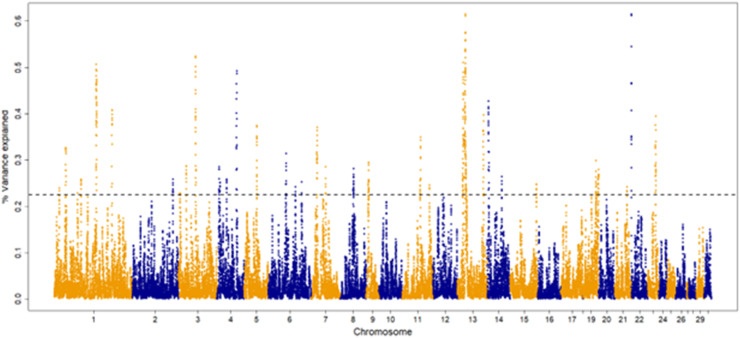
Manhattan plot for percentage of variance explained by a 30-SNP sliding window across the genome for turkey tender weight (kg). The top 1 % of SNP windows that explain the most genetic variance are located above the horizontal dashed line.

**Fig. 3:**
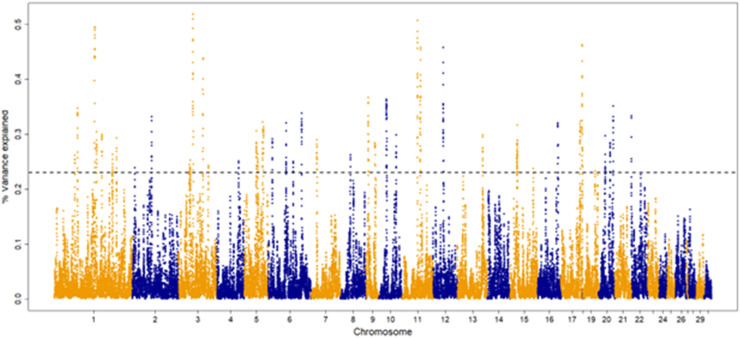
Manhattan plot for percentage of variance explained by a 30-SNP sliding window across the genome for turkey thigh weight (kg). The top 1 % of SNP windows that explain the most genetic variance are located above the horizontal dashed line.

**Fig. 4:**
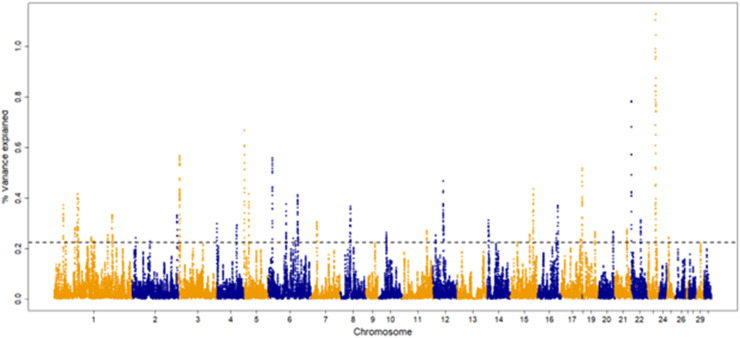
Manhattan plot for percentage of variance explained by a 30-SNP sliding window across the genome for turkey drum weight (kg). The top 1 % of SNP windows that explain the most genetic variance are located above the horizontal dashed line.

The percentage of genetic variance explained by each 30-SNP sliding window for each trait are shown in Fig. 1, Fig. 2, Fig. 3, Fig. 4. The amount of genetic variance explained by each window ranged from 0.2 × 10^-6^ - 1.3 %, 0.1 × 10^-^ - 0.6 %, 0.04 × 10^-6^ - 0.52 % and 0.2 × 10^-6^ - 1.1 % for fillets, tenders, thighs and drums, respectively. This resulted in 82, 92, 109 and, 103 positional candidate genes identified for fillets, tenders, thighs and drums, respectively.

The positional candidate genes’ corresponding KEGG pathways were identified and are shown in Table 3, biological processes in Table 4, cellular components in Table 5, and molecular function in Table 6. Positional candidate genes that were associated with a KEGG pathway and a GO term (biological processes, molecular function, or cellular component) were further investigated as functional candidate genes (**FCG**). These FCG for each carcass portion are presented and discussed below.

**Table 3:** List of KEGG metabolic pathways significantly associated with the positional candidate genes for fillets (N_PCG_=82), tenders (N_PCG_=92), thighs (N_PCG_=109), and drums (N_PCG_=103).

Description	Gene Names	Pathway ID	*p*-value
*Fillets*			
Toll- like receptor signaling pathway	TBK1; TAB2*	gga04620	0.037580
*Tenders*			
Biotin metabolism	HLCS*	gga00780	0.019431
Retinol metabolism	CYP26A1*; CYP26C1	gga00830	0.022574
Arachidonic acid metabolism	CBR1*; PTGS1	gga00590	0.038518
*Thighs*			
MAPK signaling pathway	TAB1*; PDGFB; IGF1*; STK3; PPP3CB; CACNA1G	gga04010	0.010901
Folate biosynthesis	GGH; GPHN	gga00790	0.016457
SNARE interactions in vesicular transport	STX11*; VAMP7*	gga04130	0.018862
*Drums*			
MAPK signaling pathway	TAB1*; PDGFB*; IGF1*; NFATC1*; PPP3CB; CACNA1G	gga04010	0.006984
Oocyte meiosis	IGF1*; ANAPC4; PPP3CB	gga04114	0.025200

*Indicates a positional candidate gene that was associated with a KEGG pathways and a significant GO term.

**Table 4:** Gene Ontology (GO) terms related to Biological Processes (BP) that were significantly associated with the positional candidate genes for the different turkey carcass portions (tenders, fillets, thighs, and drums).

GO ID	GO Term	p-value	Gene Names
*Fillets*			
GO:0043588	skin development	0.005649	LATS1;WDR48;TP63
GO:0006575	cellular modified amino acid metabolic process	0.009804	IYD;MTHFD1L;PCYOX1L
GO:1904888	cranial skeletal system development	0.013690	MTHFD1L;TP63
GO:0006790	sulfur compound metabolic process	0.039254	GNS;CHST9;PCYOX1L
GO:0001501	skeletal system development	0.044890	MTHFD1L;CSRNP1;WDR48;TP63
*Tenders*			
GO:0006766	vitamin metabolic process	0.017305	CBR1;CYP26A1
GO:0031099	regeneration	0.032799	STK24;MSTN
GO:0019725	cellular homeostasis	0.042973	SLC34A2;SLC40A1;ORMDL1;MSTN;TRPM7
GO:0007264	small GTPase mediated signal transduction	0.046380	DOCK9;FARP1;MCF2L2;RALGPS1
*Thighs*			
GO:0006812	cation transport	0.000963	KCNJ15;ATP5PF;PDGFB;KCNS2;ATP6V1D;SLC25A29;ANO10;SLC40A1;VAMP7
GO:0048017	inositol lipid-mediated signaling	0.006567	PDGFB;IGF1;SLC9A3R1
GO:0043491	protein kinase B signaling	0.006887	IGF1;MSTN;SLC9A3R1
GO:0051338	regulation of transferase activity	0.008545	DNAJC3;TAB1;PDGFB;IGF1;MSTN;DCUN1D1;SLC9A3R1
GO:0033002	muscle cell proliferation	0.011458	PDGFB;IGF1;MSTN
GO:0048144	fibroblast proliferation	0.014647	PDGFB;IGF1
GO:0051093	negative regulation of developmental process	0.018530	GABPA;PDGFB;IGF1;TTPA;MSTN;TP63
GO:0034220	ion transmembrane transport	0.018969	KCNJ15;ATP5PF;KCNS2;ATP6V1D;SLC25A29;ANO10;SLC40A1
GO:0070997	neuron death	0.022864	IGF1;EIF2S1;TP63
GO:0007167	enzyme linked receptor protein signaling pathway	0.028096	TAB1;PDGFB;IGF1;CSRNP1;MSTN;SLC9A3R1
GO:0043269	regulation of ion transport	0.031435	KCNJ15;PDGFB;KCNS2;VAMP7
GO:0030031	cell projection assembly	0.032548	CBY1;ATP6V1D;MSTN;TENM2
GO:0031099	regeneration	0.035730	IGF1;MSTN
GO:0051130	positive regulation of cellular component organization	0.041840	PDGFB;IGF1;KATNBL1;MSTN;VAMP7;TENM2
GO:0048284	organelle fusion	0.046484	STX11;VAMP7
GO:0033365	protein localization to organelle	0.046922	KDELR3;IGF1;ATP6V1D;VAMP7;KPNA2
Drums			
GO:0031099	regeneration	0.000173	STK24;IGF1;MSTN;MYOZ1
GO:0009611	response to wounding	0.000450	STK24;PDGFB;IGF1;SMOC2;MSTN;MYOZ1
GO:0061061	muscle structure development	0.000456	LMOD2;PDGFB;CBY1;IGF1;NFATC1;MSTN;MYOZ1
GO:0048285	organelle fission	0.004712	PDGFB;DMC1;IGF1;TRIP13;CTDP1
GO:0048017	inositol lipid-mediated signaling	0.005763	PDGFB;IGF1;SLC9A3R1
GO:0043491	protein kinase B signaling	0.006045	IGF1;MSTN;SLC9A3R1
GO:0007167	enzyme linked receptor protein signaling pathway	0.006209	TAB1;PDGFB;IGF1;SMOC2;CSRNP1;MSTN;SLC9A3R1
GO:0072348	sulfur compound transport	0.009151	SLC1A4;SLC9A3R1
GO:0033002	muscle cell proliferation	0.010084	PDGFB;IGF1;MSTN
GO:0048144	fibroblast proliferation	0.013401	PDGFB;IGF1
GO:0051270	regulation of cellular component movement	0.014923	STK24;PDGFB;IGF1;SMOC2;MSTN;SLC9A3R1
GO:0032101	regulation of response to external stimulus	0.015795	STK24;PDGFB;SMOC2;MSTN;MYOZ1
GO:0040012	regulation of locomotion	0.016009	STK24;PDGFB;IGF1;SMOC2;MSTN;SLC9A3R1
GO:0060537	muscle tissue development	0.017701	CBY1;IGF1;MSTN;MYOZ1
GO:0040007	growth	0.023674	IGF1;SERTAD2;ADNP2;WDR48;MSTN;MYOZ1
GO:0051338	regulation of transferase activity	0.023965	STK24;TAB1;PDGFB;IGF1;MSTN;SLC9A3R1
GO:0010648	negative regulation of cell communication	0.028892	CBY1;IGF1;NFATC1;SFRP2;MSTN;MYOZ1;SLC9A3R1
GO:0023057	negative regulation of signaling	0.029183	CBY1;IGF1;NFATC1;SFRP2;MSTN;MYOZ1;SLC9A3R1
GO:0006812	cation transport	0.036939	SLC9A2;PDGFB;SLC1A4;KCNG2;SLC34A2;SLC40A1
GO:0016311	dephosphorylation	0.037086	TAB1;CTDP1;MYOZ1;PPP2R2B
GO:0072359	circulatory system development	0.038527	TAB1;PDGFB;CBY1;IGF1;SMOC2;NFATC1
GO:0003012	muscle system process	0.040031	LMOD2;IGF1;MYOZ1
GO:0019725	cellular homeostasis	0.042973	SLC9A2;SLC34A2;SLC40A1;ORMDL1;MSTN
GO:0023014	signal transduction by protein phosphorylation	0.043483	STK24;TAB1;PDGFB;IGF1;MSTN

**Table 5:** Gene Ontology (GO) terms related to Cellular Components (CC) that were significantly associated with positional candidate genes for turkey carcass portion weights (fillets, thighs, and drums)*.

GO ID	GO Term	p-value	Gene Names
*Fillets*			
GO:0030496	midbody	0.004214506	KATNA1;DTNBP1;CTDP1
GO:0012506	vesicle membrane	0.023732807	IYD;DTNBP1;VAMP7
GO:0044433	cytoplasmic vesicle part	0.044096308	WDR91;IYD;DTNBP1;VAMP7
*Thighs*			
GO:0098858	actin-based cell projection	0.011724316	UTRN;TENM2;SLC9A3R1
GO:0031201	SNARE complex	0.015097471	STX11;VAMP7
GO:0042175	nuclear outer membrane-endoplasmic reticulum membrane network	0.015973273	KDELR3;DEGS2;ORMDL1;VAMP7;DIO1;LRRC59
GO:0016469	proton-transporting two-sector ATPase complex	0.021377609	ATP5PF;ATP6V1D
GO:0031984	organelle subcompartment	0.024918715	KDELR3;DEGS2;ORMDL1;VAMP7;TMEM59;DIO1;B3GNT5;LRRC59
GO:0044432	endoplasmic reticulum part	0.027625863	KDELR3;DEGS2;ORMDL1;VAMP7;DIO1;LRRC59
GO:0030133	transport vesicle	0.044557518	STX11;SLC40A1;VAMP7
*Drums*			
GO:0005581	collagen trimer	0.018351577	C1QB;C1QA

⁎ No significant CC terms were identified for the tenders portion.

**Table 6:** Gene Ontology (GO) terms related to Molecular Functions (MF) that were significantly associated with positional candidate genes identified from the turkey carcass portions (tenders, fillets, thighs)*.

GO ID	GO Term	p-value	Gene Names
*Fillets*			
GO:0003682	chromatin binding	0.022765	GABPA;JARID2;NFATC1;TP63
GO:0003700	DNA-binding transcription factor activity	0.026554	GABPA;ESR1;JARID2;NFATC1;CSRNP1;TP63
GO:0140030	modification-dependent protein binding	0.026696	TAB2;JARID2
*Tenders*			
GO:0051020	GTPase binding	0.007109	DOCK9;FARP1;MCF2L2;RABGAP1;RALGPS1
GO:0044877	protein-containing complex binding	0.025162	MYOM1;IST1;DAB2IP;NDUFA8;MRRF;CRB2
GO:0019842	vitamin binding	0.044405	HLCS;RBP4
GO:1901681	sulfur compound binding	0.069192	HLCS;MSTN
*Thighs*			
GO:0050839	cell adhesion molecule binding	0.016442	IGF1;UTRN;TENM2
GO:0000149	SNARE binding	0.031728	STX11;VAMP7
GO:0022857	transmembrane transporter activity	0.037655	KCNJ15;ATP5PF;KCNS2;ATP6V1D;SLC25A29;ANO10;SLC40A1

⁎ No significant MF terms were identified for the tenders portion.

#### Fillets

TAB2 (TGF-beta activated kinase 1/MAP3K7 binding protein 2) is involved in regulating inflammation. This gene was the only FCG identified for the fillet portion. Related to meat quality, TAB2 has been associated with shear force measurements in beef cattle (Arikawa et al., 2024). In poultry studies, TAB2 has been found to be associated with immune challenges. In broiler chickens, exposure to hydrogen sulfide gas increased the expression of TAB2 due to its involvement in necroptosis (Chi et al., 2019). Increased expression of TAB2 was also found in broiler chickens treated with probiotics compared to control birds fed a basal diet (Rajput et al., 2017). The inflammatory response is known to be associated with poultry breast meat myopathies like woody breast (Xing et al., 2021). Xing et al. (2021) demonstrated that the NK-kB signaling pathway was enhanced in breast fillets affected with woody breast and TAB2 has been previously reported to be involved in the activation of this signalling pathway (Ishitani et al., 2003). Although woody breast is less common in turkeys, the white striping myopathy is more common and varying severities occurred in the studied population (Vanderhout et al., 2024). Both myopathies are associated with increased levels of inflammation (Che et al., 2022; Prisco et al., 2021) and are known to vary with breast meat yield (Vanderhout, et al., 2022) which may explain the appearance as an FCG. In addition to implications for meat quality, TAB2 is also associated with osteoclast activity (Kim et al., 2018) and thus may be related to skeletal size. Although the breast fillets in this study were deboned, the size of the fillet is likely associated with the overall size and weight of the animal.

#### Tenders

Three FCG were identified (CYP26A1, CBR1, and HLCS) for the tenders. Cytochrome p450 family 26 subfamily A polypeptide 1CYP26A1 is a gene associated with catabolism, or the oxidation into inactive forms, of vitamin A (Zhou et al., 2012). Vitamin A is an essential fat-soluble vitamin that has key roles in reproduction and immune system regulation (Long et al., 2011). In other species, CYP26A1 has been demonstrated to be crucial for regulating vitamin A levels and supporting normal embryo development. Vitamin A supplementation has been shown to influence broiler chicken breast fillet weight (Savaris et al., 2021), which was explained by vitamin A’s role in cellular development and proliferation (Blomhoff et al., 1990). Moreover, CYP26A1 also has a function in the gut which may be associated with nutrient absorption and growth (Huang et al., 2025).

CBR1 is a carbohydrate metabolic process gene which has been reported to be associated with fat deposition in chickens (Claire D’Andre et al., 2013). This gene has been found to be down-regulated in the hypothalamus of slow-growing chickens compared to fast growing chickens (Claire D’Andre et al., 2013). Although the birds in this study were all males, it is possible the association with fat deposition arose due to the positive association between fat deposition and egg production (Melnychuk et al., 1997), given that two out of three lines used in this study were female lines. CBR1 was also identified as a differentially expressed gene in a study comparing affected and unaffected chicken breast fillets with woody breast. It was found that this gene was downregulated in fillets expressing the myopathy, potentially due to its involvement in necrosis and cell death (Bottje et al., 2021).

HLCS is the gene encoding the holocarboxylase synthetase family of enzymes involved in the cellular protein modification process by promoting biotin utilization to regulate gluconeogenesis, fatty acid synthesis, and branched chain amino acid catabolism (Yin et al., 2019). Yin et al. (2019) identified HLCS in a whole-genome sequencing study to identify selection signatures in Pengxian Yellow chickens. The finding of HLCS, in this study, as a gene under selection was attributed to biotin supplementation in the poultry industry to promote growth, which may also explain why this gene was associated with tender weights. Qi et al. (2024) also identified HLCS as a candidate gene in a GWAS of serum biochemical parameters in ducks to be associated with cholesterol, bilirubin, and low- and high-density lipoprotein concentrations. The involvement of this gene in the protein modification experience may explain why it was identified as a candidate gene for tender weight. In a study of turkeys, HLCS was also identified as a gene targeted by differentially expressed micro-RNA in muscle satellite cells in response to thermal challenge (Reed et al., 2017).

#### Thighs

For the thigh portion, the FCGs were IGF1, PDGFB, TAB1, STX11 and VAMP7. TAB1, PDGFB, and IGF1 are associated with MAPK signalling. IGF1 is the gene encoding insulin-like growth factor 1 which is associated with numerous metabolic and anabolic processes (McMurtry et al., 1997). The involvement of IGFs in the regulation of body and muscle growth in poultry is well-established. In broiler chickens, several studies have reported that certain polymorphisms of the IGF1 gene have been associated with improved growth, increased breast muscle weight, decreased abdominal fat, and enhanced skeletal integrity (Bhattacharya et al., 2015; Pandey et al., 2013; Zhou et al., 2005). However, an association between IGF1 polymorphisms and body weight was not found in a study of a locally adapted Nigerian turkey population (Oyewola et al., 2018).

TGF-β activated kinase-1 binding protein-1 (TAB1) is part of a group that regulates cell growth and differentiation (Komatsu et al., 2002), as well as associated with signalling pathways in the immune response. This gene encoding TAB1 was upregulated in chicken embryo cells infected with infectious bronchitis leading into increased cytokine production (Zhang et al., 2024). Upregulation of TAB1 has also been associated with resistance to *Campylobacter jejuni* in broiler chickens by enhancing the immune response and preventing colonization of the bacteria (Li et al., 2011).

PDGF (platelet-derived growth factor) is a family of genes and receptors involved in angiogenesis and steroid hormone production (McDerment et al., 2015). PDGFB is a growth-related gene that has been differentially expressed in the liver and spleen of chickens treated with an anti-inflammatory dietary supplement (Park et al., 2013). PDGFB has also been found to promote the proliferation of satellite cells in an *in vitro* experiment with mouse myogenic cells (Smith et al., 1999). The gene expression of PDGFB was downregulated in a study of Pekin ducks deficient in threonine, which affected breast muscle growth (Jia et al., 2023). Due to the involvement of satellite cells in post-hatch muscle growth, it is not surprising that PDBGF is associated with portion weights.

The last two FCG identified in the thighs were Syntaxin 11 (STX11) and Vesicle-associated membrane protein 7 (VAMP7) which associated with the SNARE family of proteins used in vesicular transport. The STX11 protein is particularly enriched in organs involved in the immune response (Prekeris et al., 2000). This gene was differentially expressed in broiler spleens treated with probiotics (Gu et al., 2020) and in laying hens infected with coccidiosis (Bacciu et al., 2014). Davoudi et al. (2024) found that this gene was associated with mink performance in the Aleutian disease test which is an immune response stimulation. The VAMP7 gene is believed to have played a role in the domestication of poultry through the modulation of behaviour (Vignal et al., 2019). Although, polymorphisms of the VAMP7 gene have been associated with differences in cattle growth which may explain its presence as a FCG in this study (Liu et al., 2023).

#### Drums

Like thighs, TAB1, PDGFB, IGF1 discussed above were identified as FCG in the drums. Additionally in the drums, NFATC1 was identified as an FCG. NFATC1 is involved in the Wnt signalling pathway with roles in negative regulation of cell communication, negative regulation of cell signalling, circulatory development, and negative regulation of developmental process. This gene was also identified as an FCG in a GWAS of turkey breast white striping which was attributed to the association between rapid muscle growth and the myopathy (Vanderhout et al., 2024). This gene was also identified as a host gene of differentially expressed circular RNAs between breast and leg muscle fibers of an indigenous Chinese chicken breed (Ju et al., 2021).

### Gene ontology

Several biological process GO terms related to muscle growth and development significantly associated with the PCGs were identified and shown in Table 3. For example, in the breast muscles (fillets and thighs) terms related to skin (GO:0043588) and skeletal system development (GO:0001501) were identified. For the legs (drums and thighs), terms related to muscle cell proliferation (GO:0033002), muscle structure (GO:0061061) and tissue (GO:0060537) development, and growth (GO:0040007). Some of these terms (i.e., GO:0061061, GO:0040007) have also been identified in other poultry studies such as that of Reed et al. (2022) who identified genes associated with the response to thermal challenge in turkey muscle satellite cells and Xiao et al. (2021) who identified differentially expressed genes between high and low fat chickens from an indigenous Chinese breed. Other terms (i.e., GO:0060537) have been reported in studies of species like swine in relation to differentially expressed genes during heat stress (Ma et al., 2019). Some of the GO terms identified in the breast meat that are less obviously connected to muscle growth (i.e., skeletal system development - GO:0001501) have been reported in other poultry studies involving phenotypic divergence between layer and broiler chicken lines (Contriciani et al., 2024). Additionally, the GO term related to skin development (GO:0043588) was identified. However, this term was reported to be associated with skeletal muscle maturation and hypertrophy-related differentially expressed genes in swine (Mohammadinejad et al., 2022). No biological process GO terms were in common between the fillets and the other three portions. However, significant biological process GO terms were shared among the other three traits. Tenders, thighs, and drums shared GO:0031099 which is related to regeneration. Thighs and drums shared 6 additional terms related to inositol lipid-mediated signaling (GO:0048017), protein kinase B signaling (GO:0043491), regulation of transferase activity (GO:0051338), muscle cell proliferation (GO:0033002), fibroblast proliferation (GO:0048144), and enzyme linked receptor protein signaling pathway (GO:0007167).

### Relationship between growth and the immune system

Many of the FCGs identified in this study were related to immune system function. Several of these genes are implicated in tissue regeneration or response to injury so it is not surprising that they were significantly associated with carcass portion weights as these are sites of major tissue growth. It is also possible that these genes were identified due to the established relationship between growth and health. Selection for rapid growth rates has been shown to result in a reduced immunological response in turkeys (Bayyari et al., 1997; Huff et al., 2005). It is known that macrophages (a key component of the immune response) play a role in muscle growth through the stimulation of satellite cells (Malila et al., 2022; Merly et al., 1999). However, to our knowledge, the association between meat yield and these aspects of the immune response have not been specifically investigated in turkeys. This area merits further study when considering designing breeding programs that do not have negative consequences for animal health, welfare, and robustness.

Overall, the present study identified genes associated with turkey carcass portion weights to provide further insight into the genetic architecture of these traits. It should be acknowledged that this study gives suggestive QTL and genes of interest and allows us to speculate on the role of these genes in the growth and development of turkey carcass portions. Further work should be done to validate these genes (i.e., divergent selection trials, gene expression studies) in turkeys. The weight of the turkey carcass portions was estimated to be highly heritable, and many of the FCGs identified were associated with inflammation and proinflammatory responses. Interestingly, there was very limited overlap between the FCGs of the different carcass portions. Biologically, we would have expected similar genes identified between the fillets and tenders (breast muscles) and thighs and drums (leg muscles). Although we would not necessarily expect there to be many similar genes between the breast and leg muscles. Any genes in common across all four traits would likely be related to overall bodyweight/size which would influence the weight of all muscles in the body, regardless of location. As reported in Vanderhout et al. (2022) using the same dataset, the genetic correlation between the fillet and tender traits was 0.45, which was higher than the correlation between the fillets and drums (0.27) and fillets and thighs (0.37). Drums and thighs had a genetic correlation of 0.85. This may illustrate why several FCGs were found in common between the thighs and drums, but none were found in common between the fillet and tenders (contrary to expectations), and none were found in common across all traits. This illuminates avenues for future studies to investigate targeted selection for different carcass portions.

## Declaration of competing interest

The authors declare the following financial interests/personal relationships which may be considered as potential competing interests:

Christine Baes and Benjamin Wood reports financial support was provided by 10.13039/100008762Genome Canada. Christine Baes and Benjamin Wood reports financial support was provided by 10.13039/501100000092Ontario Genomics Institute. Christine Baes reports financial support was provided by 10.13039/501100000038Natural Sciences and Engineering Research Council of Canada. Christine Baes and Benjamin Wood reports financial support was provided by Hybrid Turkeys. If there are other authors, they declare that they have no known competing financial interests or personal relationships that could have appeared to influence the work reported in this paper.
